# *Hemiboeachanii* (Gesneriaceae), a new species from limestone areas of northern Vietnam

**DOI:** 10.3897/phytokeys.183.69180

**Published:** 2021-10-22

**Authors:** Cuong Huu Nguyen, Ly Van Nguyen, Khang Sinh Nguyen, Alexander A. Egorov, Leonid V. Averyanov

**Affiliations:** 1 Vietnam National University of Forestry, Xuan Mai, Chuong My, Ha Noi, Vietnam Vietnam National University of Forestry Ha Noi Vietnam; 2 Saint Petersburg State Forestry University, Institute str., 5, St. Petersburg, 194021, Russia Saint Petersburg State Forestry University St.Petersburg Russia; 3 Institute of Ecology and Biological Resources, Vietnam Academy of Science and Technology, 18 Hoang Quoc Viet Road, Nghia Do, Cau Giay, Hanoi, Vietnam Institute of Ecology and Biological Resources, Vietnam Academy of Science and Technology Ha Noi Vietnam; 4 St. Petersburg State University, 7-9 Universitetskaya Emb., St. Petersburg, 199034, Russia St. Petersburg State University St.Petersburg Russia; 5 Komarov Botanical Institute Russian Academy of Sciences, Prof. Popov str., 2, St. Petersburg, 197376, Russia Komarov Botanical Institute of the Russian Academy of Science St. Petersburg Russia

**Keywords:** Endemism, flora of Vietnam, limestone flora, New taxon, plant diversity, plant taxonomy

## Abstract

*Hemiboeachanii*, a new species of Gesneriaceae from Ha Giang Province, northern Vietnam, is here described and illustrated. It has many branched stems, diamond-shaped involucre with two cirrose opposite apices, a pink corolla, red spotted inside, and a flowering time in January-February. Among congeners with an externally hairy corolla, this new species is morphologically close to *H.crystallina* and *H.sinovietnamica*. Diagnostic discriminative characters in all mentioned species are discussed. The conservation status of this species is considered to be “Critically endangered” (CR) according to the IUCN Red List Categories and Criteria.

## Introduction

Prior to 2011, *Hemiboea*Clarke (1798) (Gesneriaceae) contained about 23 species and 5 varieties, distributed mainly from northern Vietnam to southern and eastern China to southern Japan (Weber 2004; Li and Wang 2004). In 2011 the formerly recognized genus, *Metabriggsia* W.T.Wang (1983), with two species, *Metabriggsiaovalifolia* W.T.Wang and *Metabriggsiapurpureotincta* W.T.Wang, was revised and merged with *Hemiboea* (Weber et al. 2011). During last two decades, 14 new taxa were found and described in China and Vietnam: 13 new species and one new variety from Guangxi, Guizhou and Yunnan provinces of southern and southwestern China (Huang et al. 2020) and one from Thanh Hoa Province of northern Vietnam (Nguyen et al. 2019a). In addition, based on extensive morphological, phenological, and molecular studies, H.subcapitatavar.pterocaulis Z.Y.Li (Li 2004) was accepted as a distinct species *H.pterocaulis* (Z.Y.Li) J.Huang, X.G.Xiang & Q.Zhang in Huang et al. (2017). Similarly, H.subcapitatavar.guangdongensis (Z.Y.Li) Z.Y.Li became *H.guangdongensis* (Z.Y.Li) X.Q.Li & X.G.Xiang in Li et al. (2019). In total, before our study, the genus *Hemiboea* comprises at least 39 species and 5 varieties.

During the botanical fieldwork in limestone areas of Ha Giang Province in northern Vietnam in spring 2021, we collected an unusual species of *Hemiboea*. The genus characteristics are calyx 5-parted to the base, stamens 2, staminodes 3, stigma 1 (undivided), and capsule oblique in relation to pedicel, narrowly lanceolate, somewhat curved, dehiscing adaxially; valves 2, straight, not twisted. After consulting relevant literature of *Hemiboea* (Wang et al. 1998; Pham 2000; Li and Wang 2004; Wei et al. 2010; Do et al. 2016; Luu et al. 2018; Wei 2018; Nguyen et al. 2019b) including recently published papers for new taxa of Gesneriaceae from Vietnam (e.g. *Michaelmoelleria* F.Wen, Y.G.Wei & T.V.Do in Wen et al. 2020) as well as the examining of herbarium specimens, we identified our plants as a new species, well segregated from all known species of *Hemiboea* by its morphological characters, namely a diamond-shaped involucre with a cirrose apex; a 1-veined calyx with flat, not revolute margin; a pink corolla with red spots, and a slightly swollen stigma. This new species is described and illustrated here as *H.chanii* C.H.Nguyen & Aver.

## Material and methods

All collected and studied specimens of the newly discovered species are presently stored in the Herbaria of China, Russia, and Vietnam (IBK, IBSC, KUN, LE, PE, VNF). The photographs used for the species illustration were taken in the species natural habitats. Morphological observations and measurements were made on living plants, dry specimens, and materials preserved in alcohol. Morphological characters are described using the terminology proposed by Wang et al. (1998).

## Taxonomic treatment

### Hemiboea chanii

Taxon classificationPlantaeLamialesGesneriaceae

C.H.Nguyen & Aver. sp. nov.

893F573D-D125-5EDB-98B7-D722A2684F79

urn:lsid:ipni.org:names:77221221-1

Figs 1 , 2

#### Type.

Vietnam. Ha Giang Province: Vi Xuyen District, Minh Tan Village, primary evergreen broad-leaved forest, around point 23°00'14.9"N 104°54'55.9"E, altitude 533 m, 19 January 2021, *N.V.Ly*, *NVL 20210119001* (holotype: VNF!; isotype: LE http://en.herbariumle.ru/?t=occ&id=91550).

#### Diagnosis.

*Hemiboeachanii* is similar to *H.crystallina* Y.M.Shui & W.H.Chen in the shape of leaf blade, leaf margin and the externally pubescent corolla, but differs in having non-inflated nodes, diamond-shaped not winged involucre, flat calyx margin, corolla red-spotted inside with a ring of hairs, and stigma slightly swollen. It also morphologically resembles *H.sinovietnamica* W.B.Xu & X.Y.Zhuang but differs in having 4–8 branches on main stem, repand-crenate leaf margin, involucre diamond-shaped with a cirrose apex, and pink corolla and a slightly swollen stigma (Table 1).

#### Description.

Perennial lithophytic herb. Stem ascending to erect, with 12–16 nodes on the main stems, not inflated, glabrous, subterete, 50–90 cm tall, 3–7 mm in diameter, with 4–8 branches. Leaves petiolate, opposite, unequal to sub-equal in a pair; petiole 1.5–4.5 cm long, about 2 mm in diameter, glabrous, green to purple; leaf blade narrowly ovate, ovate, oblong or elliptic, coriaceous when dry, 7.5–12 cm long, 2.5–5 cm wide, glabrous, adaxial surface dark green, abaxial surface pale greenish, at base cuneate, sometimes slightly oblique, apex acute or shortly acuminate, repand-crenate along the margin, median and lateral veins inconspicuous adaxially and protuberant abaxially, lateral veins in 6–10 pairs. Inflorescence subterminal, 2–3-flowered cyme; peduncle 0.8–1 cm long, about 1 mm in diameter, glabrous, green to pale green; involucre diamond-shaped, not winged, 1.6–1.8 cm in diameter, outside glabrous, with two cirrose opposite apices. Calyx white, actinomorphic, 5-lobed, dissected from the base; segments subequal, narrowly lanceolate 1.3–1.4 × 0.2–0.3 cm, glabrous, margin entire flat, 1-veined. Corolla infundibular, outside pink with numerous red spots on adaxial lip, inside red spotted, 3.5–4 cm long; tube 3.0–3.6 cm long, 1.4–1.6 cm in diameter at the orifice, 3–4 mm in diameter at the base, sparsely glandular pubescent outside; inside with a ring of hairs adnate to 3–4 mm above the corolla base; limb distinctly two-lipped; adaxial lip 3–4 mm long, 2-lobed at apex, lobes subequal, nearly semi-circular, margin recurved; abaxial lip 6–8 mm long, 3-lobed, lobes unequal, with rounded apex, median lobe larger, broadly ovate, lateral 2 smaller, slightly obliquely ovate. Stamens 2, adaxial, adnate to 1.1–1.3 cm above the corolla base; filaments filiform, coiled, 1.2–1.4 cm long, 1 mm in diameter; anthers basifixed, globular, 1.5–1.7 mm in diameter, coherent at the apex. Staminodes 3, linear, glabrous, with inflated apex, adnate to 13–15 mm above the corolla base, the middle one 2–3 mm long, lateral ones 9–10 mm long. Disc circular, lemon-yellow, 1.2–1.4 mm high, margin repand, glabrous. Pistil 24–26 mm long; ovary narrowly cylindrical, glabrous, 7–8 mm long, about 2 mm in diameter; style 17–18 mm long, terete, glabrous, about 1 mm in diameter, apex curved; stigma slightly swollen, truncate. Capsule oblique in relation to pedicel, terete to narrowly fusiform, 2.6–3 cm long, glabrous, slightly curved, dehiscing adaxially; valves 2, straight, not twisted.

**Figure 1.**
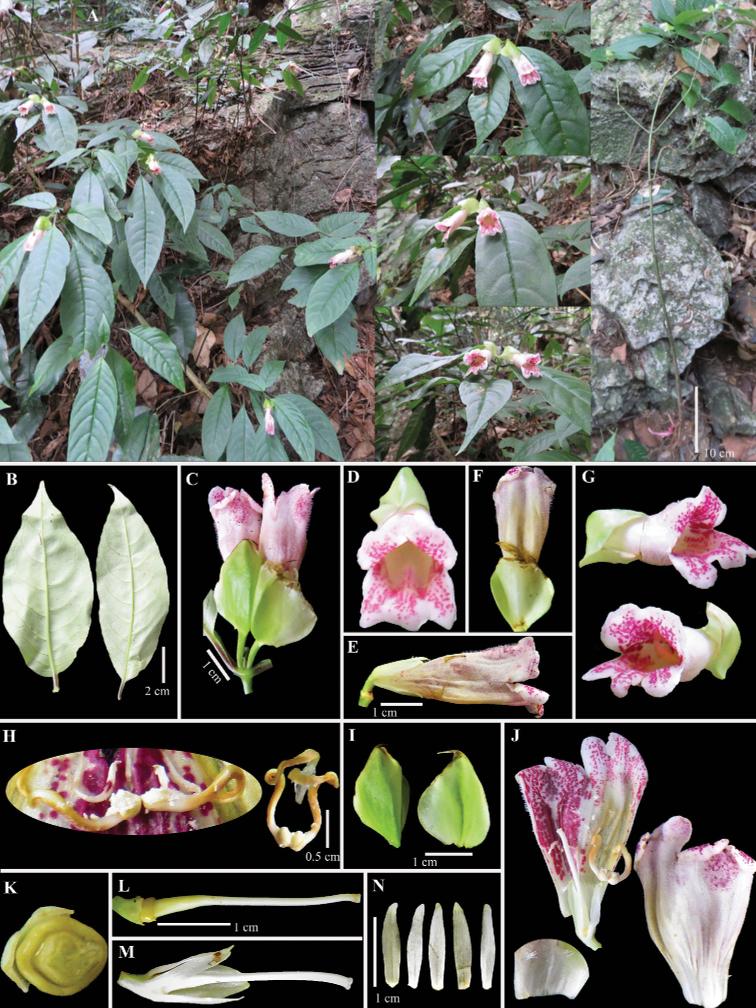
*Hemiboeachanii* C.H.Nguyen & Aver., sp. nov. **A** plants in natural habitat **B** leaf blade, abaxial surface **C** flowering branch **D** flower, frontal view **E–G** flower, side and half side views **H** stamens and staminodes **I** involucre **J** flower inside and outside views **K** ripening capsule, cross section **L-M** pistil and ripening capsule, side view **N** calyx segments. Photos by Nguyen Van Ly, correction and design by C.H. Nguyen.

**Figure 2.**
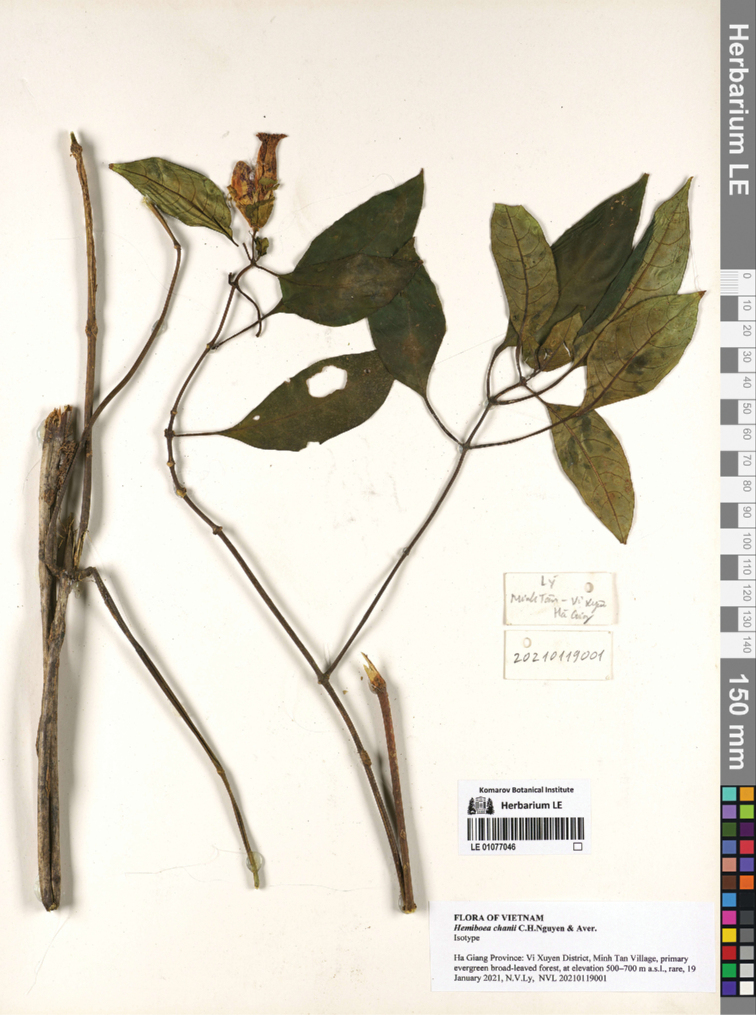
*Hemiboeachanii* C.H.Nguyen & Aver., sp. nov. isotype specimen detail. N.V.Ly, NVL 20210119001 (isotype: LE 01077046 http://en.herbariumle.ru/?t=occ&id=91550).

#### Distribution and habitat.

The new species is only known from Minh Tan Village, Ha Giang Province, growing in cracks of limestone rocks in moist shady places or occasionally in the upper part of slopes, and on rocky hilltops in primary evergreen broad-leaved forests on karstic limestone at elevations 500–700 m a.s.l. Some of main herbaceous species accompanying the new species have been recorded as *Aglaonemamodestum* Schott ex Engl., *Amorphophallustonkinensis* Engl. & Gehrm., *Begoniaspp*., *Impatiensbonii* Hook.f., *Laporteainterrupta* (L.) Chew, *Primulinabalansae* (Drake) Mich.Möller & A.Weber, and *Rhaphidophoradecursiva* (Roxb.) Schott

#### Phenology.

Flowering from January to February, and fruiting from February to April.

#### Etymology.

*Hemiboeachanii* is named in honor of the lecturer, Mr. Le Mong Chan, for his outstanding contributions to the conservation of the flora of Vietnam.

#### Proposed IUCN conservation status.

At present, only one known population with less than 100 mature individuals is confirmed in the field assessment. Its estimated area of occupancy is less than 5 km^2^. The population and habitat are greatly susceptible to various human activities and damage, particularly forest logging, agriculture, and grazing. There is a high risk of habitat degradation in the future because it is located close to the local village. Following the IUCN Red List Categories and Criteria (IUCN 2017), the new species may be assessed tentatively as Critically Endangered [B2ab (ii, iii), CR].

#### Note.

*Hemiboeachanii* is morphologically similar to *H.crystallina* and *H.sinovietnamica* in its ecology. It has only been found in a limestone area and grows in the evergreen broad-leaved forest. The new species can be easily distinguished from *H.crystallina* in having a subterete stem up to 90 cm tall (vs. stem terete up to 40 cm tall), non inflated nodes (vs. nodes inflated), 1-veined calyx with flat, not revolute margin (vs. calyx 3-veined with revolute margin), corolla red spotted inside with a ring of hairs (vs. corolla inside purplish-red spotted with pale yellowish lines and absence of hair ring), and stigma slightly swollen (vs. stigma not swollen). The new species differs from *H.sinovietnamica* in having a subterete, 4–8 branched stem up to 90 cm tall (vs. stem subtetragonal, simple, up to 45 cm tall), repand-crenate leaf margin (vs. entire leaf margin), involucre diamond-shaped with a cirrose apex (vs. involucre triangular with acute apex), corolla pink (vs. corolla yellowish), stigma slightly swollen (vs. stigma distinctly capitate), and a flowering period lasting in January-February (vs. flowering period lasting during August-October). The comparison of the key morphological characters of *H.chanii*, *H.crystallina*, and *H.sinovietnamica* is presented in Table 1.

**Table 1.** Morphological characters of *H.chanii*, *H.crystallina* and *H.sinovietnamica*.

	*H.chanii*	*H.crystallina*	*H.sinovietnamica*
Stem height and shape of cross-section	50–90 cm, subterete	40 cm or less, terete	25–45 cm, subtetragonal
Number of stem branches	4–8	2–6	stem simple
Node	not inflated	inflated	not inflated
Leaf margin	repand-crenate	repand-crenate	entire
Involucre characters	diamond-shaped, not winged, apex cirrose	quadrangular, winged on costas, apex acute	triangular, not winged, apex acute
Calyx lobe characters	margin flat, not revolute, 1-veined	margin revolute, 3-veined	margin flat, not revolute, 1-veined
Corolla characters	pink, inside red spotted with a ring of hairs	white, inside purplish-red spotted with pale yellowish lines, hair ring absent	yellowish, inside purple-spotted with a ring of hairs
Stigma	slightly swollen	slightly obtuse	distinctly capitate
Flowering period	January–February	November	August–October

## Supplementary Material

XML Treatment for Hemiboea chanii
